# Controlled human infection models in COVID-19 and tuberculosis: current progress and future challenges

**DOI:** 10.3389/fimmu.2023.1211388

**Published:** 2023-05-25

**Authors:** Hazel Morrison, Susan Jackson, Helen McShane

**Affiliations:** Jenner Institute, University of Oxford, Oxford, United Kingdom

**Keywords:** tuberculosis, COVID-19, CHIM, controlled human infection, challenge models

## Abstract

Controlled Human Infection Models (CHIMs) involve deliberately exposing healthy human volunteers to a known pathogen, to allow the detailed study of disease processes and evaluate methods of treatment and prevention, including next generation vaccines. CHIMs are in development for both tuberculosis (TB) and Covid-19, but challenges remain in their ongoing optimisation and refinement. It would be unethical to deliberately infect humans with virulent *Mycobacteria tuberculosis* (*M.tb*), however surrogate models involving other mycobacteria, *M.tb* Purified Protein Derivative or genetically modified forms of *M.tb* either exist or are under development. These utilise varying routes of administration, including *via* aerosol, per bronchoscope or intradermal injection, each with their own advantages and disadvantages. Intranasal CHIMs with SARS-CoV-2 were developed against the backdrop of the evolving Covid-19 pandemic and are currently being utilised to both assess viral kinetics, interrogate the local and systemic immunological responses post exposure, and identify immune correlates of protection. In future it is hoped they can be used to assess new treatments and vaccines. The changing face of the pandemic, including the emergence of new virus variants and increasing levels of vaccination and natural immunity within populations, has provided a unique and complex environment within which to develop a SARS-CoV-2 CHIM. This article will discuss current progress and potential future developments in CHIMs for these two globally significant pathogens.

## Introduction

**Figure 1:**
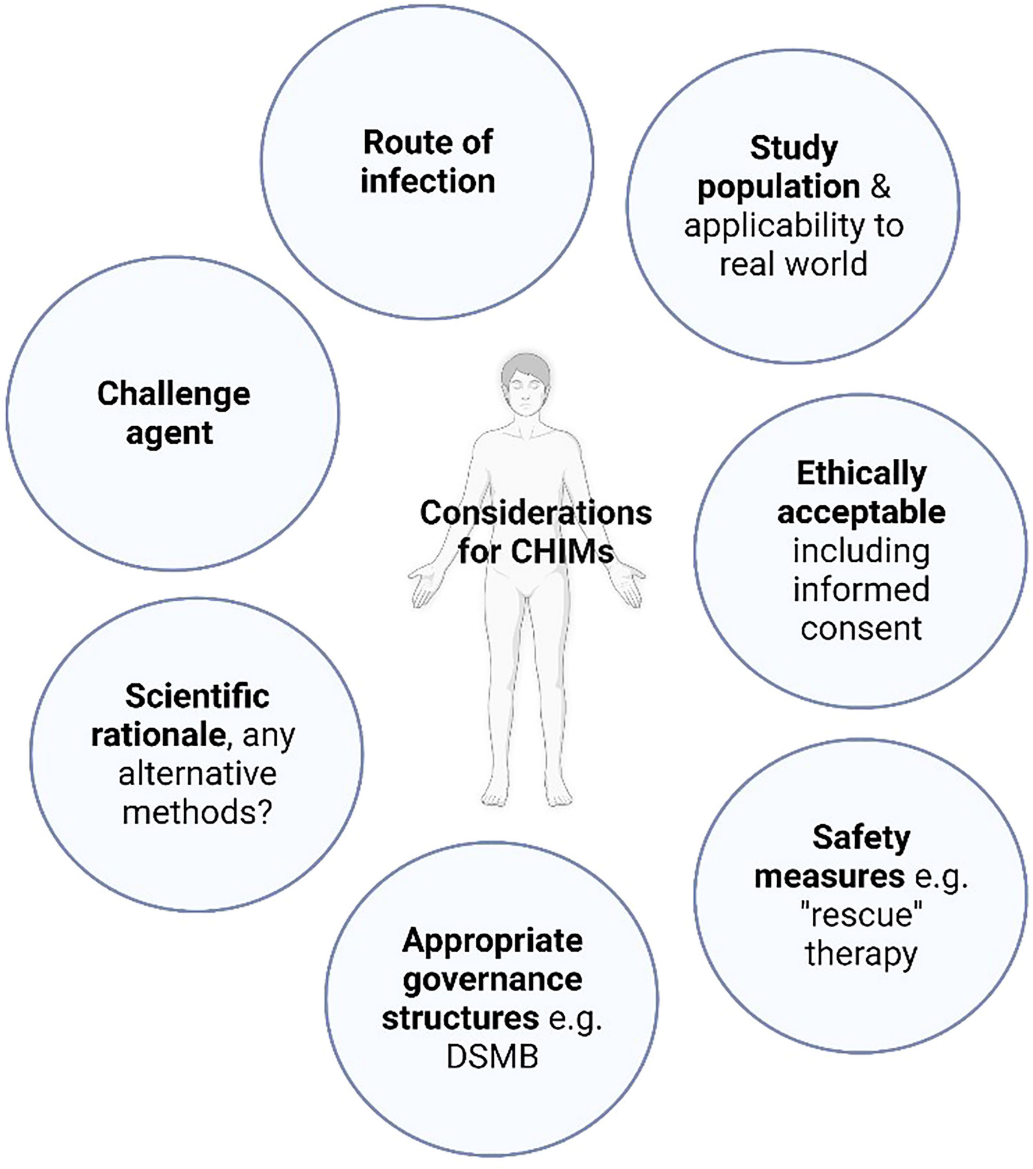
Controlled human infection model design. A common framework of considerations for CHIM design should be employed. “Rescue” therapy: treatment employed in CHIMs either to prevent the progression of volunteer symptoms experienced beyond mild disease or to abrogate infection, DSMB: Data safety monitoring board.

Controlled human infection models (CHIMs) involve the deliberate inoculation of volunteers with a pathogen under carefully controlled conditions, facilitating detailed study of host-pathogen immunobiology. Validated models can then be used to expedite the development of novel vaccines and therapeutics by allowing efficacy testing in small scale clinical trials, prior to field efficacy studies. Dating back to Edward Jenner’s 18^th^ century smallpox experiments, historically, the ethical conduct of CHIMs has been controversial. With the implementation of modern ethical frameworks and considered study design (**Figure 1**), they have proven to be a safe and efficacious tool, particularly in the field of vaccinology, contributing to the development of vaccines for malaria, influenza, typhoid and cholera (1–4).

Tuberculosis (TB) remains a major global health issue, second only to COVID-19 as the leading cause of death from a single infectious pathogen (5). The COVID pandemic has itself reversed decades of progress towards meeting global TB reduction targets and new tools to combat TB are urgently needed (6, 7). Whilst astonishing research efforts worldwide have rapidly led to multiple licensed COVID-19 vaccines and therapeutics (8, 9), the ongoing potential of the virus to mutate, coupled with changing population immunity, means we cannot be complacent in our quest to develop new scientific tools and evaluate next generation vaccines and treatments. CHIMs against these two different, but both highly consequential, respiratory pathogens could be harnessed to help accelerate progress.

## Tuberculosis controlled human infection models

### Background and need for a TB CHIM

The only licenced vaccine against TB, Bacillus-Calmette Guérin (BCG), provides good protection against severe forms of infant TB, but highly variable efficacy against pulmonary TB and therefore limited impact on disease transmission. Ongoing challenges also exist in the accurate diagnosis of both TB infection and active disease, increasing drug resistance and treatment burden even for fully sensitive disease (10). Despite huge research efforts, developments in all of these areas are hampered by gaps in our understanding of intricate host-pathogen interactions, the complex spectrum of disease states that cannot be replicated fully in animal models and lack of defined immune correlates of protection (CoP). Judicious use of a mycobacterial CHIM could help facilitate advances in many of these domains, as a complement to animal and field studies (11). For example, a mycobacterial CHIM could enable the prioritisation of vaccine candidates that most effectively control mycobacterial growth, prior to larger, more costly field efficacy studies. Samples from such a CHIM could also be used to interrogate immune parameters that correlate with control after a defined timepoint infection, with any positive steps towards finding a validated TB immune CoP proving potentially transformative.

### Current and future approaches to developing a TB CHIM

Intentionally infecting humans with virulent *Mycobacterium tuberculosis* (*M.tb*) would not be ethical, with the potential for significant morbidity and mortality. Even if these are avoided, long treatment duration with the risk of significant drug side effects, risk of *M.tb* transmission to others, inability to prove cure at the end of treatment and possibility of disease recurrence are all substantial arguments against a CHIM with wild-type *M.tb*. Therefore, researchers must pursue the use of alternative challenge agents (Summarised in **Table 1**), aiming to address key scientific questions with an acceptable risk profile to both volunteers and the wider community.

**Table 1:** Controlled Human Challenge Studies in Tuberculosis and Covid-19.

Studies, country	Challenge agent, *route*	Challenge overview	Key findings	Challenge method comments
Tuberculosis				
Tomlinson et al. 2011. UK, South Africa (12).	PPD 5 TU *ID*	Volunteers with a spectrum of mycobacterial exposure underwent concurrent TSTs in each arm, with skin biopsies at 6 and 48 hours	• Recruitment of TH1-polarised responses and cytotoxic T-cells at TST site • Immune responses predominantly due to cell recruitment, not proliferation	**ID PPD** **✓** Minimally invasive **✓** Allows dissection of immune responses and interactions *in vivo* **✗** Not at site of natural infection **✗** Unable to assess vaccine/ therapeutic efficacy as no replication
Pollara et al. 2017. South Africa, UK, Peru (13).	PPD 5 TU *ID*	PPD or saline control injection in individuals with active TB, LTBI or cured disease, followed by skin biopsy of TST site at 48 hours	• Elevated levels of IL17A/F and enrichment of Th17 cells in active TB compared to LTBI • Associated with increased neutrophils and MMP-1 • Changes reversed in cured group	
Silver et al. 2003. USA (14).	PPD 0.01-0.5 TU *Intrabronchial*	Dose escalation study of PPD instillation per bronchoscope followed by bronchoalveolar lavage (BAL) at 48 hours in TST positive and negative individuals	• Local inflammatory response at 0.5 TU, with increased mobilisation of CD4+ T-cells and antigen-specific IFNγ producing cells in the lungs of TST positive volunteers	**Intrabronchial PPD** **✓** Allows dissection of immune responses and interactions *in vivo* **✓** At mucosal site of natural infection **✗** Invasive instillation and sampling **✗** Unable to assess vaccine/ therapeutic efficacy as no replication
Walrath et al. 2005. USA (15).	PPD 0.5 TU *Intrabronchial*	Follow on PPD per bronchoscope study to examine mucosal immune responses by BAL 48 hours after installation in TST positive and negative individuals	• IFN-γ-inducible chemokines including CXCR3 ligands increased in TST individuals • Evidence of compartmentalised resident memory cell induction	
Schreiber et al. 2010. UK (16).	BCG Moreau 10^7^ viable bacilli *Oral*	Repeated oral challenge days 0, 28, 49 in historically BCG vaccinated volunteers with subsequent peripheral blood sampling	• Increase in PPD-specific IFNγ seen 6 months after 1st challenge • Increase in IL6-enriched pathways at day 7, no changes after repeat challenge	**Oral BCG** **✓** Non-invasive **✓** Live, replicating organism **✓** Challenge involves mucosal site **✗** Not natural site of TB infection **✗** Study designed as surrogate CHIM for gastrointestinal infections not TB **✗** Mucosal sampling difficult **✗** Minimally immunogenic **✗** Difficult/unable to quantify viable BCG
Minassian et al. 2012. UK (17).	BCG Danish 1331 1-4×10^5^ CFU *ID*	Feasibility study of ID BCG challenge in BCG-naïve and historically vaccinated. Skin biopsies and suction blisters used to quantify BCG recovery and examine cellular infiltrate	• Peak BCG recovery in challenge site at 2 weeks (detectable up to 4 weeks) • CD15+ neutrophilic infiltration at blister site • Prior BCG vaccination lead to reduction in recoverable BCG by PCR	**ID BCG** **✓** Same route as vaccination, same safety profile **✓** Live, replicating organism **✓** Minimally-invasive (skin biopsies) **✓** Easily controllable and quantifiable **✓** Proven to detect a BCG vaccine effect **✗** Cannot be used to study vaccines based on RD1 deleted antigens **✗** Not at natural site of infection **✗** Unable to assess involvement of respiratory mucosal immunity in control
Harris et al. 2013. UK (18).	BCG Danish 1331, 2-8x10^5^ CFU *ID*	Use of ID BCG CHIM to asses vaccine candidate MVA85A prime or as a booster following historical BCG-vaccination, with skin biopsies taken 2 weeks following BCG challenge	• Protective BCG vaccine effect again detectable by PCR • No added benefit of MVA85A over BCG (in keeping with field trials)	
Minhinnick et al. 2016. UK (19).	BCG Danish 1331 or BCG TICE, standard ( 2-8 x10^5^ CFU) or high (3 x standard) *ID*	Optimisation of ID BCG challenge model by BCG strain and dose in BCG-naïve volunteers, with skin biopsies taken 2 weeks following BCG challenge	• No significant difference in BCG recovery by strain • High dose ID BCG was well tolerated with improved BCG recovery • High-dose BCG Danish 1331 identified as optimal agent for future studies	
Blazevic et al. 2017. USA (20).	BCG TICE 2x10^6^ *ID*	ID BCG challenge to asses use of skin swabs to detect BCG	• BCG detection possible *via* swabs, but less reproducible and consistent than biopsy • Later recovery of BCG (3-4 weeks)	
Davids et al. 2020. South Africa (21).	BCG Danish 1331 1x10^3^-1x10^5^ CFU *Intrabronchial* Also PPD 0.2 TU and 0.5 TU *Intrabronchial*	Safety and feasibility study with per bronchoscope instillation of BCG and PPD (different lung segments) in volunteers with a broad range of prior mycobacterial sensitisation	• Highly compartmentalised immune responses demonstrated, localised to the challenged lung segments • Frequency of Th17 homing cells unexpectedly seen to decrease after PPD or BCG challenge	**Intrabronchial BCG** **✓** At mucosal site of natural infection **✓** Allows dissection of pulmonary and systemic immune responses *in vivo* **✓** Live, replicating organism **✓** Safety shown in sensitised individuals **✗** Cannot be used to study vaccines based on RD1 deleted antigens **✗** Invasive challenge **✗** Invasive sampling **✗** Accurate quantification of BCG recovery from pulmonary samples challenging **Intrabronchial PPD** See comments on previous studies
TB041, UK. Clinicaltrials.gov NCT02709278 Completed, manuscript under review (11)	BCG Danish 1331 /BCG Bulgaria 1×10^3^ - 1×10^7^ CFU *Aerosol inhaled*	Dose escalation study of aerosol BCG in BCG-naïve volunteers, with comparison ID BCG am	• Aerosol BCG is safe, and immunogenic in BCG naïve volunteers • Live BCG can be detected from BAL samples	**Aerosol BCG** **✓** At mucosal site of natural infection **✓** Non-invasive challenge, most closely mimics natural inoculation **✓** Allows dissection of immune responses and interactions *in vivo* **✓** Live, replicating **✗** Cannot be used to study vaccines based on RD1 deleted antigens **✗** Invasive sampling **✗** Accurate quantification of BCG recovery from pulmonary samples challenging
TB043, UK. Clinicaltrials.gov NCT03912207 Ongoing (22)	BCG Danish 1331 1X10^7^ CFU *Aerosol inhaled*	Exploratory study into innate and adaptive immune response to aerosol mycobacterial challenge	Trial protocol only, results awaited	
TB044, UK. Clinicaltrials.gov NCT04777721, Ongoing (22)	BCG Danish 1331 1x10^4^ - 1 ×10^7^ *Aerosol inhaled*	Dose escalation study of aerosol BCG in historically BCG vaccinated volunteers	Trial protocol only, results awaited	
COVID-19/ SARS-CoV-2				
Killingley et al. 2022. UK (23).	Wild-type SARS-CoV-2 virus (SARS-CoV-2 /human/GBR/ 484861/2020) 10 TCID_50_ *Intranasal*	Dose finding study, healthy 18-30-year olds, seronegative with no prior SARS-CoV-2 infection or vaccination	• 18/34 (53%) of volunteers developed productive infection at 10 TCID50 • Accurate description of 1^o^ infection viral kinetics with pre-Alpha strain • Virus detectable in throat significantly earlier than the nose, but reaching higher titres in nose • Viral shedding started at 2 days post inoculation and peaked at 5 days at 8.87log10 copies per millilitre • Viable virus was detected on FFA for an average of 10 days (up to 12 days) • Challenge was safe and well tolerated. No evidence of lower respiratory tract infection but 83% of volunteers demonstrated measurable smell disturbance. • Strong correlation with lateral flow positivity and viable virus on FFA	**Wild type, seronegative** **✓** Proof of concept, ethical acceptability and safety **✓** Able to establish productive infection **✓** Able to dissect primary infection kinetics and immune response under standardised conditions **✗** No longer dominant variant **✗** Seronegative model cannot be used for vaccine/ therapeutic development given global seroprevalence *via* vaccination/ infection
COV-CHIM01, UK. Clinicaltrials.gov NCT04864548, Ongoing	Wild-type SARS-CoV-2 virus (SARS-CoV-2 /human/GBR/ 484861/2020) 10-1x10^5^TCID_50_ *Intranasal*	Dose finding study, healthy 18-30-year olds, prior SARS-CoV-2 infection +/- vaccination. Utilising same pre-Alpha SARS-CoV-2 virus as seronegative CHIM. Starting at dose of 1x10^1^ up to 1x10^5^TCID50.	Trial protocol only, results awaited	**Wild type, seropositive** **✓** Seropositive studies needed for real world utility **✗** Potent protection against re-infection demonstrated in field studies prior to emergence of Delta & Omicron variants ?feasibility (results awaited)
COVHIC002, UK. ISRCTN94747181, Ongoing	Delta SARS-CoV-2 virus Starting dose 1x10^2^ TCID_50_ *Intranasal*	Dose finding study, healthy 18-30-year olds, SARS-CoV-2 vaccinated (+/- prior infection).	Trial protocol only, results awaited	**Viral variants, seropositive** **✓** Seropositive studies needed for real world utility **✓** Use of variants allows dissection of heterologous immunity **✓** More reflective of real world **✓** Proof of concept with viral variants could allow selection of optimum challenge strain for future use ✗ Viral mutation likely to outpace GMP manufacture of challenge strains **✗** Potent protection against re-infection demonstrated in field studies prior to emergence of Omicron variant ?feasibility

**✓** Positive aspect of model **✗** Drawback to model.

BAL, bronchoalveolar lavage; BCG, Bacillus-Calmette Guérin; ID, intradermal; FFA, focus forming assay; IL6, interleukin 6; IL17, interleukin 17; LTBI, latent TB infection; MMP-1, matrix metalloproteinase-1; PPD, tuberculin purified protein derivative, TCID_50_, median tissue culture infectious dose; Th17, T-helper 17; TST, tuberculin skin test; TU, tuberculin Unit.

The tuberculin skin test (TST), where tuberculin Purified Protein Derivative (PPD) is injected intradermally, has traditionally been used as a diagnostic test for latent TB infection (LTBI). It has been employed as a challenge agent to investigate immunological responses to mycobacterial antigens at the site of skin challenge, for example identifying exaggerated Th17 responses in those with active TB disease as a potential target for host directed therapies (12, 13, 24). PPD has also been used to assess local respiratory mucosal responses following intrabronchial instillation (14, 15, 21). Whilst these methods may contribute to our knowledge of mycobacterial immunopathogenesis they cannot be utilised directly to assess efficacy of vaccines or therapeutics.

A CHIM that is to be used to evaluate vaccine efficacy requires a live replicating organism, for example an attenuated strain of mycobacteria. BCG itself is such a live attenuated mycobacteria, initially derived *via* passage from *Mycobacterium bovis (M. Bovis)*, that does not cause disease or latency in healthy humans (25). The loss of key virulence genes encoded in the Region of Difference 1 (RD1) during this process confers the advantageous safety profile of BCG but means the full immunopathogenic pathways of *M.tb* are not entirely replicated and it could not be used to evaluate vaccines which incorporate RD1-encoded antigens, such as ESAT-6 and CFP-10. However, BCG has been shown to induce similar canonical CD4^+^ T cell-mediated immune responses to *M. tb* in humans (26) and assessment of vaccine efficacy using a BCG challenge in animal models are comparable to results obtained using *M.tb* as the challenge agent (27, 28). BCG manufactured under good manufacturing practice (GMP) conditions for human use is readily available and this therefore represents the only live replicating TB CHIM agent currently available (11).

CHIMs using intradermal (ID) BCG as a mycobacterial challenge agent have been developed and are able to detect a known BCG vaccine effect in animals and humans (17, 18, 27–29). The ID route allows straightforward quantification of mycobacteria from an easily accessible site, for example via minimally invasive punch skin biopsies (17). However, the natural route of *M.tb* infection is via the respiratory tract and initial pathogen interactions with the specialised host respiratory mucosal system cannot be evaluated using an ID CHIM.

Efforts are ongoing to develop pulmonary CHIMs that more closely mimic the natural route of *M.tb* infection. BCG delivered via aerosol (Clinicatrials.gov NCT02709278, NCT03912207, NCT04777721) or instilled directly into the lungs per bronchoscope (21) are both being evaluated and have been shown to be safe and well tolerated. A defined timepoint pulmonary mycobacterial infection would allow examination of localised mucosal immunology and the relationship to induced system responses, which are key areas of research interest. Vaccines or therapeutics tested using these CHIMs would have the advantage of accounting for the contribution of the specialised respiratory mucosa in conferring protective immunity. However, sampling of the respiratory mucosa for immunological interrogation and quantification of recoverable BCG in pulmonary models are both more complex and invasive than in skin models (30).

Following on from initial studies using BCG, live mycobacterial CHIMs could be enhanced by the use of rationally attenuated genetically modified organisms. BCG which has been modified, for example to include a fluorophore reporter gene or exhaled volatile compound detectable by mass spectrometry could reduce the need for invasive sampling for mycobacterial recovery and quantification (31, 32). Use of current live vaccine candidates such as MTBVAC, a rationally attenuated form of *M.tb* (33, 34) or VPM1002, a recombinant BCG (35, 36), could allow investigation of the antigens or immunological pathways missing from BCG.

Whilst it is some way off from clinical evaluation, efforts are underway to develop a conditionally replicating *M.tb* strain with a genetically inserted suicide mechanism. This would aim to recapitulate the initial immunological mechanisms of *M.tb*, whilst ensuring complete eradication at a predefined timepoint and, if successful, could hugely advance the field of human TB study (22, 31).

Finally, for a TB CHIM to be truly useful, it should be safe, acceptable and deliverable in TB endemic populations and settings. Different environmental exposures, level of nutrition, microbiome composition, prior exposure to mycobacteria and prevalence of co-infections are just some of the known factors impacting vaccine efficacy. Utilising an ethically appropriate CHIM in endemic settings would ensure vaccines are tested in relevant populations (11, 37).

## SARS-CoV-2 controlled human infection models

### Background and need for a SARS-CoV-2 CHIM

Early in the COVID-19 pandemic, the World Health Organisation (WHO) acknowledged the potential benefits of a SARS-CoV-2 CHIM, for example to allow rapid prioritisation of vaccine candidates. A working group was promptly established to consider the practicalities, feasibility and ethics (38). Initial expert consensus was divided with concern about the lack of a suitable “rescue” therapy, potential for severe illness and high transmissibility, as well as the benefit and applicability of such a model over field studies (39).

Accruing data suggested that infection of young, healthy adults in whom disease was generally much milder could be justifiable. This prompted UK manufacture of a challenge virus under GMP conditions and development and rigorous ethical review of study protocols for both a UK SARS-CoV-2 naïve CHIM (NCT04865237) and one in previously infected volunteers (NCT04864548) (40). GMP manufacture of challenge viruses is a time-consuming process and enrolment did not commence in these studies until March (NCT04865237) and May, 2021 (NCT04864548) respectively, by which point several highly efficacious vaccine candidates were being deployed in the UK population (41, 42).

Despite the widespread availability of highly effective vaccines against SARS-CoV-2, there remains a justifiable role for SARS CoV-2 CHIMs. A clear advantage of a CHIM over natural infection field studies is the known-timepoint of infection; allowing the detailed characterisation of both viral kinetics and the host immune response post-exposure. The dose of virus can also be carefully controlled and adjusted, providing crucial information about how the infectious dose affects the clinical and immunological response to the virus. Importantly, CHIMs also allow the collection of pre-exposure samples. These baseline samples can be assessed against clinical outcomes to identify immune correlates of protection (CoP).

Whilst current literature clearly defines the role of neutralising antibodies (nABs) as a correlate for sterilising immunity against SARS-CoV-2 (43–46), emerging evidence, particularly with the evolution of Variants of Concern (VoC) that escape nABs, is that the immune response to SARS-CoV-2 is more complex. Cell-mediated immunity, memory B cells and non-neutralising Fc-mediated effector functions may all play a role (47–53). Local mucosal immune responses have demonstrably protected against infection from other respiratory pathogens (54, 55) but mucosal immunity against SARS-CoV-2 remains poorly described in the literature. A CHIM with infection at a controlled timepoint allows the detailed interrogation of all aspects of the protective immune response, particularly the early host mucosal responses that are often missed in natural field infection studies.

Furthermore, the ability to control confounders such as inoculum strain, route of exposure, viral load and patient heterogeneity in a CHIM allows direct comparison of vaccine and therapeutic candidates as well as dosing regimens. With the roll-out of successful vaccines, it is unfeasible and unethical to maintain an unvaccinated placebo group for the testing of new vaccine candidates. Non-inferiority trials require large sample sizes and sufficient naturally acquired infection which can be time consuming and expensive. A CHIM could be of particular use in assessing novel vaccines, including those developed to be mucosally-delivered, which may have differing end-points (such as prevention of infection or viral shedding) that would be extremely difficult to study without a defined timepoint of infection. Whilst field studies are considered gold standard for vaccine licensure, there are instances where CHIMs have been used directly as proof of efficacy (4).

### Current and future approaches to developing a SARS-CoV-2 CHIM

To date there are three registered SARS-CoV-2 CHIMs (Summarised in **Table 1**). The wild-type (pre-Alpha) SARS-CoV-2 CHIM in healthy, seronegative, UK 18-29-year olds demonstrated infection in 53% (18/34) of volunteers using a low inoculum dose of 10TCID50 (50% tissue culture infectious dose). Challenge was safe and well-tolerated with no evidence of lower respiratory tract involvement, although smell disturbance was common and prolonged in a small number of volunteers (23). Killingley et al. were able to accurately delineate the viral kinetics of primary infection and identified differences in viral dynamics depending on swab site. Viable virus measured by focus forming assay (FFA) persisted for on average 10 (maximum of 12) days, consistent with pre-Alpha isolation guidance (23). FFA was shown to closely correlate with lateral flow antigen (LFA) tests performed on the same swab samples. This first in human SARS-CoV-2 CHIM has demonstrated the broad utility of CHIMs, strengthening confidence in the public health measures (such as isolation periods and use of LFA tests) employed in the UK. Exploration of immune correlates of protection in this seronegative cohort, such as cross-reactive responses from seasonal coronaviruses, is ongoing.

With increasing global seroprevalence to SARS-CoV-2 from vaccination and/or infection (56), a seropositive SARS-CoV-2 CHIM is needed in order to facilitate future vaccine and therapeutic development in volunteers that reflects real world immunity. Successfully establishing a re-infection model additionally allows the identification of both local and systemic immune markers attained via the infection or vaccination process that are protective against re-infection, which could inform future public health strategies as well as design of therapeutics and vaccines.

Ongoing use of a pre-Alpha strain for a seropositive CHIM has several potential issues. Field data suggests that acquired immunity (either by vaccination, natural infection or both – hybrid immunity) offers strong resistance to homologous re-infection (57, 58). Achieving consistent infection rates may therefore prove more difficult than in a study of naïve participants.

Much of knowledge of re-infection rates was obtained prior to the emergence of variants such as Delta and subsequently Omicron, which are known to escape immunity. Both variants have antigenic divergence due to mutations in the spike protein and have been shown to demonstrate reduced neutralisation titres compared to pre-Alpha strains in vaccinated and hybrid cohorts (59–62). One approach which may circumvent any difficulty achieving infection in seropositive volunteers is to use variants more likely to cause breakthrough infections as the challenge agent, such as the Delta variant (isrctn.com ISRCTN94747181). Manufacture of an Omicron challenge agent is also being pursued (63).

There are pros and cons to the use of Delta or Omicron in a CHIM. Neutralisation against the Omicron variant is more markedly reduced than delta and associated with a higher rate of breakthrough infections (47, 61, 64) making it plausible that it would be easier to achieve infection in a CHIM. Omicron may also be a safer challenge agent demonstrating milder disease severity and reduced lower respiratory tract disease (65–68). However, the shorter infection course seen with the Omicron variant may also make it difficult to assess post-infection therapeutics (69).

Studies using currently prevalent variants are arguably more relevant both for the development pipeline of vaccines and therapeutics and understanding CoP. Limitations to this approach are that manufacturing a new challenge strain under GMP conditions takes at least 6 months (70). Furthermore, any specific clinical risks of that variant need to be understood from real world data prior to use in an ethically sound CHIM. The high incidence of SARS-CoV-2 and associated viral replication globally has resulted in the relatively rapid acquisition of mutations and development of new VoCs, meaning that by the time an inoculum strain is ready for use in a CHIM it may no longer be the dominant variant in the real world. However, developing several CHIMs that use variants derived from different lineages will enable broad assessment of different therapeutics and vaccines.

## Discussion

Tuberculosis and Covid-19 represent two deadly, but distinct, respiratory diseases. Whilst highly efficacious vaccines against Covid-19 were developed at unprecedented speed against the backdrop of the evolving pandemic, progress in improving on the limited overall efficacy of the BCG vaccine against TB has been much slower. All possible research approaches that can be utilised to expedite progress should be harnessed to improve this situation. We must also remain vigilant against the potential for further SARS-CoV-2 mutations and need to have methods available to be able to rapidly assess new vaccines and therapeutics.

CHIMs may prove to be useful tools in our armoury against both of these pandemic pathogens, despite their unique situations and challenges. There are no validated CoP in TB and use of CHIMs to interrogate human immunological responses following a defined timepoint infection could increase our understanding in this area. Whilst validated CoP, for example in the form of nABs, do exist for Covid-19, these are clearly not the only factor contributing to immunity, particularly against initial infection and transmissibility. The early host mucosal immune response to *M.tb* and SARS-CoV-2 represent an important knowledge gap for both pathogens. The ability to abort infection at its point of entry could prevent LTBI and provide epidemic control by blocking onward transmission of SARS-CoV-2. These initial mucosal responses can only really be studied in an experimental setting with a known timepoint of infection.

Identification of the ideal challenge agent for a CHIM remains an issue for both of these diseases. Use of virulent *M.tb* is unethical and therefore any deployable TB CHIM will only provide partial information about the true protective efficacy of a tested vaccine or therapeutic against *M.tb*. Progress is underway to identify surrogate agents which could be utilised and, given the differing advantages and disadvantages of various agents and routes of challenge (see **Table 1**), it may be that a combination of the available options will need to be employed depending on the exact question to be answered or until new modified organisms are available (11). In Covid-19, viral mutations mean that manufacture of a challenge agent may lag behind currently circulating variants. Utilisation of a variant with optimal challenge properties (for example, high levels of infectivity with low potential to cause severe disease), such as those seen in the Omicron variant may be one approach. Or it may be that, similarly to TB, a range of challenge agents could be developed and utilised depending on the specific question to be answered.

A CHIM for the purpose of novel vaccine and therapeutic evaluation needs to be able to accurately quantify pathogen load. This is undertaken with quantitative PCR (qPCR) on minimally invasive samples from the oral or nasal mucosa for SARS-CoV-2. The sampling and quantification of mycobacteria, particularly from the respiratory tract, for a TB CHIM remains much less straightforward, for example due to the fastidious and slow growing nature of mycobacteria and colonisation of the respiratory tract with organisms including non-tuberculous mycobacteria. One potential entirely non-invasive solution under development is the use of specially adapted face masks, containing a collection matrix to sample exhaled pathogens, which are then detected *via* qPCR. Initially developed as a potential diagnostic tool for TB (71), these are currently being evaluated in both TB (Clinicaltrials.gov NCT03912207) and COVID (Clinicaltrials.gov NCT04864548), highlighting how solutions initially designed for one pathogen can be utilised in another.

Applicability of CHIMs utilised in young, healthy adults to real world populations of interest is an area of consideration for both pathogens. In TB, there is a drive to deliver CHIMs in TB endemic settings, to ensure information derived and interventions tested are relevant to eventual target populations (11). In Covid-19, applicability of results obtained in a CHIM to those most at risk of disease, including the elderly and immunocompromised, is not yet known. There may be fundamental differences in the way these populations respond to the virus that limit the generalisability of a CHIM conducted purely in young, immunocompetent adults. Interestingly, in more established respiratory pathogens, efforts are underway to develop safe CHIMs in older adults (72), but it is not at all clear that this would be ethical or feasible with SARS-CoV-2

With multiple studies ongoing to develop and optimise CHIMs within both TB and Covid-19, this is an area of considerable scientific interest and promise. Momentum gained in research during the Covid-19 pandemic should be harnessed to ensure CHIMs for these, and other, pathogens continue to be developed and to exploit their full potential, in particular the fields of vaccine development and to further our understanding of host-pathogen immunobiology.

## Author contributions

HMo and SJ contirubuted equally to this work and share first authorship. HMo and SJ wrote the first draft of the manuscript. All authors contributed to the article and approved the submitted version.
